# Small intestinal bacterial overgrowth mimicking acute flare as a pitfall in patients with Crohn's Disease

**DOI:** 10.1186/1471-230X-9-61

**Published:** 2009-07-30

**Authors:** Jochen Klaus, Ulrike Spaniol, Guido Adler, Richard A Mason, Max Reinshagen, Christian von Tirpitz C

**Affiliations:** 1 University Medical Center Ulm, Center for Internal Medicine, Department of Internal Medicine I, Germany; 2 University Hospitals of Cleveland Case Medical Center, Case Western Reserve University School of Medicine, Cleveland, Ohio, USA; 3 Klinikum Braunschweig, Department of Internal Medicine I, Salzdahlumer Straße 90, 38126 Braunschweig, Germany; 4 Medizinische Klinik, Kreisklinik Biberach, Ziegelhausstraße 50, 88400 Biberach, Germany

## Abstract

**Background:**

Small intestinal bacterial overgrowth (SIBO) is characterized by excessive proliferation of colonic bacterial species in the small bowel. Potential causes of SIBO include fistulae, strictures or motility disturbances. Hence, patients with Crohn's Disease (CD) are especially predisposed to develop SIBO. As result, CD patients may experience malabsorption and report symptoms such as weight loss, watery diarrhea, meteorism, flatulence and abdominal pain, mimicking acute flare in these patients.

**Methods:**

One-hundred-fifty patients with CD reporting increased stool frequency, meteorism and/or abdominal pain were prospectively evaluated for SIBO with the Hydrogen Glucose Breath Test (HGBT).

**Results:**

Thirty-eight patients (25.3%) were diagnosed with SIBO based on positive findings at HGBT. SIBO patients reported a higher rate of abdominal complaints and exhibited increased stool frequency (5.9 vs. 3.7 bowel movements/day, p = 0.003) and lower body weight (63.6 vs 70.4 kg, p = 0.014). There was no correlation with the Crohn's Disease Activity Index. SIBO was significantly more frequent in patients with partial resection of the colon or multiple intestinal surgeries; there was also a clear trend in patients with ileocecal resection that did not reach statistical significance. SIBO rate was also higher in patients with affection of both the colon and small bowel, while inflammation of the (neo)terminal ileum again showed only tendential association with the development of SIBO.

**Conclusion:**

SIBO represents a frequently ignored yet clinically relevant complication in CD, often mimicking acute flare. Because symptoms of SIBO are often difficult to differentiate from those caused by the underlying disease, targeted work-up is recommended in patients with corresponding clinical signs and predisposing factors.

## Introduction

The small bowel represents a transition zone between the stomach, which harbors low numbers of microorganisms, and the colon, with its high level of bacterial colonization. Small intestinal bacterial overgrowth (SIBO) represents an increased colonization by bacterial species derived from the colonic flora that may spread beyond the small bowel segments and sometimes affect the stomach. Under physiological conditions, the proximal jejunum is characterized by a bacterial population corresponding to 10^3^-10^4 ^colony forming units (CFU) per milliliter of aspirate. SIBO is characterized by a population corresponding to >10^5 ^CFU/ml of aspirate [1-3].

Direct confirmation of SIBO is possible only by microbiological examination of aspirate from the proximal small bowel [1,4]. Because of the difficulty of integrating this method into clinical routine, it has become standard procedure to utilize indirect non-invasive methods for detecting SIBO based on changes caused by the metabolic activity of bacteria colonizing these bowel segments.

Hydrogen Breath Tests (HBT) determine the concentration of hydrogen gas (H_2_) in exhaled air following the oral administration of carbohydrates. H_2 _is produced exclusively by bacteria; in healthy individuals, significant numbers of such bacteria are found only in the colon. H_2 _gas formed in the bowel passes by diffusion into the capillary circulation of the intestinal mucosa. Because of its low solubility in blood, it is completely eliminated through the lungs in exhaled air. This production of H_2 _by intestinal bacteria following oral application of carbohydrates can be used diagnostically to demonstrate, among other entities, bacterial overgrowth of the upper gastrointestinal tract with H_2_-forming microorganisms. A simple and sensitive test for detecting bacterial overgrowth is the H_2 _glucose breath test (HGBT). Physiologically absorbed carbohydrates such as glucose undergo fermentation mediated by bacteria prior to their absorption in the small bowel. A by-product of this process is H_2_. An increase in the H_2 _concentration in exhaled air (>10 ppm) over baseline (H_2 _level prior to substrate intake) after oral application of 50 mg of glucose is considered evidence of SIBO [1,3-5].

As a consequence of maldigestion and inflammatory changes of the bowel mucosal membrane secondary to SIBO, patients experience a reduction in intestinal absorption. Malabsorption affects the intake of important nutrients, like fat-soluble vitamins, and certain minerals, such as calcium. SIBO also results in a constellation of symptoms including watery diarrhea, weight loss, meteorism, flatulence and abdominal pain [1,5,6].

Factors promoting the development of SIBO include changes in the acid secretion of the stomach, fistulae, strictures and motility disturbances. In addition, an intact ileocecal valve serves as a physiological barrier between the small bowel and colon [1,7-9].

For these reasons, patients with Crohn's Disease (CD) may exhibit a predisposition for SIBO.

There is a paucity of data in the literature regarding the prevalence of SIBO in patients with CD [8-10]. In many cases, bacterial overgrowth may be overlooked due to the fact that the symptoms of SIBO do not differ significantly from those of the underlying disease process itself sometimes even mimicking an acute flare.

This, in turn, may have as a consequence that therapeutic measures, such as the administration of topical or systemic steroids, may be instituted based on the potentially incorrect assumption that patients' symptoms are the result of increased disease activity. On the other hand, SIBO can be treated with a short course of antibiotic therapy [2,11-13].

Objective of the present prospective study was to investigate the prevalence of small intestinal bacterial overgrowth (SIBO) and possible predisposing factors in a selected group of patients with CD and increased stool frequency and/or meteorism. Patients underwent H_2 _glucose breath test (HGBT) to screen for SIBO and provided information on their medical history using a standardized questionnaire.

## Methods

### Patients

Two-hundred sixty-seven consecutive CD patients attending the Crohn's/Ulcerative Colitis Outpatient Clinic of the University Hospital of Ulm received a written invitation to participate in this study. Inclusion criteria included endoscopically or histologically confirmed CD and clinical symptoms characterized by at least one of the following symptoms: diarrhea (more than four bowel movements per day), meteorism (lasting for more than two weeks) and/or abdominal pain (occurring at least one to two times per week, severity described as at least "moderate"). Exclusion criteria included use of antibiotics, therapy with probiotics, history of bowel preparation for colonoscopy within four weeks preceding study inclusion, treatment of a stoma and abdominal complains such as nausea and vomiting or abdominal colic suspected to be related to bowel stricture or obstruction. Of the total 267 invited patients, 150 patients (56.2%) fulfilled the inclusion criteria. Eighty patients did not meet the inclusion criteria, while the remaining 37 patients refused to participate in the study without citing reasons. Disease activity was assessed on the basis of the Crohn's Disease Activity Index (CDAI; [14]). Patients provided data on the clinical course of their disease using a standardized questionnaire; missing data were supplied from the patients' medical records.

The study was conducted with the approval of the ethics committee of the University of Ulm in accordance with the principles of the Helsinki Declaration and the rules of Good Clinical Practice. Written informed consent was obtained from each patient prior to inclusion into the study.

### H_2 _Glucose Breath Test (HGBT)

The H_2 _Glucose Breath Test (HGBT) was performed on an outpatient basis between 8:00 and 11:00 a.m. Patients were asked to abstain from all food for at least 12 hours prior to the test and to avoid high-carbohydrate meals on the previous day. In addition, patients were asked not to smoke for at least two hours prior to the test and to avoid physical exertion. The first breath sample was taken as baseline level. Thereafter, patients were given 50 grams of glucose in 200 ml of uncarbonated mineral water. The concentration of hydrogen in exhaled air was then measured every twenty minutes using a H_2 _analyzer (GMI Medical Ltd., Renfrew, Scotland, UK). The duration of the test was 180 minutes. An increase of more than 10 parts per million (ppm) over baseline in at least three samples within the measurement period was taken as evidence of bacterial overgrowth in the small bowel [12].

### Statistics

Results were evaluated descriptively. Continuous data were represented as median with range. Because of the non-normal distribution of continuous data within the subgroups with and without evidence of SIBO, the Mann-Whitney test was used as a non-parametric method. Differences between groups were considered statistically significant at values of *p *< 0.05. A comparison of frequencies within the subgroups was performed using a four-field test. Again, statistical significance was assigned for values of *p *< 0.05.

## Results

Patient demographics for all 150 study participants are given in table 1. There was no statistically significant difference between males and females in terms of age and average duration of the disease. As expected, males, on average, exhibited significantly greater heights and weights; however, they were comparable in terms of body-mass index (BMI; table 1).

**Table 1:** Demographic data of the 150 patients included in the study

	All	(n = 150)	Females	(n = 91)	Males	(n = 59)
Age	41	(18–72)	40	(18–72)	43	(21–71)
Body weight (kg)	68.7	(43–125)	64.5	(43–125)	75.2	(48–104)
Height (cm)	169.9	(142–192)	165.0	(142–180)	177.4	(160–192)
BMI (kg/m^2^)	23.7	(17–39)	23.6	(17–39)	23.9	(17–32)
Disease duration (years)	11.7	(0.5–36)	10.7	(0.5–33)	13.3	(0.5–36)

The diagnosis of small intestinal bacterial overgrowth (SIBO) was made on the bases of the H_2 _Glucose Breath Test (HGBT) in 38 patients (25.3%). HGBT results were negative in the remaining 112 patients.

Due to the degree of clinical symptoms during the HGBT, three patients with negative HGBT findings underwent the H_2 _Lactulose Breath Test (HLBT). Based on the results of HLBT, these three patients were classified as non-hydrogen producers. For the purposes of the present study, these patients continued to be assigned to the group without evidence of SIBO.

The groups of patients with and without SIBO did not differ in terms of age or height. The average weight of patients with SIBO, however, was significantly lower (63.6 kg vs. 70.4 kg, p = 0.014). Calculation of BMI also showed lower values for the patient group with SIBO, but the difference was not statistically significant (table 2).

**Table 2:** Age, body weight and height, and duration of disease in relation to results of the H_2_-Glucose breath test.

	SIBO (n = 38)		No SIBO (n = 112)		*p*
Age	41	(21–62)	41	(18–72)	0.887
Body weight (kg)	63.6	(43–100)	70.4	(43–125)	0.014
Body height (cm)	167.9	(147–188)	170.5	(142–192)	0.174
BMI (kg/m^2^)	22.5	(17–32)	24.1	(17–39)	0.064
Disease duration (years)	13.6	(0.75–31)	11.1	(0.5–36)	0.113

There was no statistically significant difference between patients with and without SIBO with respect to mean time since first diagnosis (table 2), although the prevalence of SIBO increased in proportion to the duration of the disease in comparison with the overall group. SIBO was observed in 18 of 97 patients (18.6%) in whom less than 15 years had elapsed since the first diagnosis of Crohn's disease, compared to 20 of 53 patients (37.7%) with a greater than 15-year history of the disease. This difference, however, remained just below the level set for statistical significance in terms of the 95% confidence interval (12.1–27.4% vs. 25.9–51.2%).

The daily stool frequency in patients with SIBO was significantly higher than in those without SIBO (5.9 vs. 3.7; p = 0.003; table 3). Stool frequency in excess of six bowel movements per day was observed in 11 patients (28.9%) with SIBO compared to only 13 patients (11.6%) in those without SIBO (p = 0.012). Low stool frequencies of less than three bowel movements per day were observed in only seven patients (18.4%) of patients with positive test results compared to 49 patients (43.8%) with negative test results (p = 0.005; table 3). By comparison, SIBO was present significantly more often in patients with a daily stool frequency of six or more bowel movements (45.8%; 95%-CI: 27.9–64.9%) than in those with less than three bowel movements per day (12.5%; 95%-CI: 6.2–23.6%; table 4).

**Table 3:** Average daily stool frequency, low, medium and high stool frequencies in patients with an without SIBO

	SIBO (n = 38)			No SIBO (n = 112)			
	*n*	*%*		*n*	*%*		*p*
< 3 stools/day	7	18.4%		49	43.8%		0.005
3–6 stools/day	20	52.6%		50	44.6%		0.394
> 6 stools/day	11	28.9%		13	11.6%		0.012
	***Median***	***95%-CI***	***Range***	***Median***	***95%-CI***	***Range***	

stools per day	5.9	+/- 1.9	1–22	3.7	+/- 0.5	1–15	0.003

**Table 4:** Prevalence of SIBO in relation to low, medium and high stool frequency

	SIBO (n = 38)		
	*n*	*%*	*95%-CI*
< 3 stools/day	7	12.5%	6.2 – 23.6%
3–6 stools/day	20	28.6%	19.3 – 40.0%
> 6 stools/day	11	45.8%	27.9 – 64.9%

Median CDAI in patients with SIBO was 166 (range: 34–406) and was only tendentially higher than in patients without SIBO (median: 139; range: 13–399; p = 0.098). Of patients with SIBO, 17 (44.7%) had a CDAI <150 and 21 (55.3%), a CDAI >150. By comparison, 65 of 112 patients (58.0%) without SIBO had a CDAI <150 and 47 patients (42%) a CDAI >150.

All participants were asked whether they experienced increased symptoms during the HGBT. Interestingly, patients with SIBO reported increased symptoms during the test significantly more often than did those without SIBO (19 [50.0%] vs. 21 [18.8%]; p = 0.0002; table 5). Reported symptoms included predominantly meteorism, diarrhea and abdominal pain. Bloating was reported significantly more often during the test in patients with SIBO (44.7% vs. 10.7%). Symptoms occurring during administration of the HGBT are given in table 5.

**Table 5:** Occurrence of symptoms and symptoms during the test in patients with and without SIBO (multiple responses possible)

	All patients (n = 150)		SIBO (n = 38)		No SIBO (n = 112)		
			*n*	*%*	*n*	*%*	*p*
Symptoms during the test			19	50,0	21	18,8	0,0002
							
	***n***	***%/95% CI***		***%/95% CI***		***%/95% CI***	

Bloating	29	19.3% 13.8 – 26.4	17	44.7% 30.2 – 60.3	12	10.7% 6.2 – 17.8	
Diarrhea	10	6.7% 3.7 – 11.8	6	15.8% 7.4 – 30.4	4	3.6% 1.4 – 8.8	
Abdominal pains	9	6.0% 3.2 – 11.0	2	5.3% 1.5 – 17.3	7	6.3% 3.1 – 12.3	
Nausea	2	1.3% 0.4 – 4.7	0	0.00% --	2	1.8% 0.5 – 6.3	
Other	4	2.6% 1.0 – 6.7	2	5.3% 1.5 – 17.3	2	1.8% 0.5 – 6.3	

The pattern of inflammation in patients in relation to SIBO is given in table 6. With respect to the pattern of inflammation with disease involving the ileum there was no significant difference between patients with and without SIBO. Patients with SIBO, however, showed a trend towards higher frequency of inflammation involving the colon and a significantly higher frequency of combined involvement of the colon and small bowel than did patients without SIBO.

**Table 6:** Pattern of inflammation and prior surgical procedures in patients with and without SIBO (multiple responses possible)

Pattern of inflammation	SIBO (n = 38)		No SIBO (n = 112)		*P*
Ileum	27	(71.1%)	72	(64.3%)	0.447
Colon	24	(63.2%)	53	(47.3%)	0.091
Stomach or jejunum	0	(0.0%)	2	(1.8%)	0.407
Colon and small bowel	14	(36.8%)	23	(20.5%)	0.044
**Surgical procedure**					
Bowel resection (all)	23	(60.5%)	56	(50.0%)	0.261
Ileocoecal resection	23	(60.5%)	48	(42.9%)	0.059
Partial colectomy	10	(26.3%)	13	(11.6%)	0.030
Multiple procedures	13	(34.2%)	16	(14.3%)	0.007

Fourteen of 37 patients (37.8%) with inflammation involving the colon and small bowel at the time of the study exhibited SIBO. SIBO was present in 24 of 77 patients (26.8%) with inflammation exclusively affecting the colon and in 27 of 99 patients (18.8%) with inflammation exclusively affecting the ileum.

Frequency and site of prior surgical procedures in relation to SIBO are given in table 6. Twenty-three of 71 patients (32.4%) who had undergone ileocecal resection tested positive for SIBO. Of the 79 patients with intact ileocecal valve, however, only 15 patients (19.0%) tested positive for SIBO (p = 0.059). Patients with prior colectomy returned pathological breath test findings in 10 of 23 cases (43.5%). By comparison, only 28 of 127 patients who had not undergone prior colectomy returned positive findings (p = 0.030). Thus, patients with prior colectomy or with multiple prior surgical procedures involving the bowel showed a significantly higher rate of SIBO; patients with prior ileocecal resection also exhibited a higher rate of SIBO, but the difference did not achieve statistical significance.

Patients with or without SIBO showed no differences in terms of their pharmacological Crohn's therapy and there was no correlation between the occurrence of SIBO and patient's current treatment with 5-aminosalicylic acid, topical or systemic steroids or immunosuppressants.

Reduced gastric acid secretion may promote bacterial growth. Of 13 patients receiving proton pump inhibitors at the time of HGBT, five returned positive findings (38.5%). Of the 137 patients who did not use acid-reducing agents, only 33 showed evidence of SIBO (24.1%; p = 0.255).

Smokers may exhibit increased H_2 _production and lead to false positive test results. Both the proportion of smokers (34.2% vs. 27.7%; p = 0.445) and their respective daily consumption of cigarettes (10.5 vs. 11.4 cigarettes per day; p = 0.473) were comparable in both groups.

## Discussion

Bacterial overgrowth of the small bowel (SIBO) has been described in association with a variety of disorders, including Crohn's disease (CD). To date, however, there is a paucity of data in the international literature addressing this complication, even though CD patients often exhibit a predisposition for SIBO. The current prospective study investigated the prevalence, predisposing factors and clinical relevance of SIBO in patients with CD. We found that fully a quarter of all patients (38/150, 25.3%) with CD and abdominal complaints suffer from SIBO.

This figure is in agreement with findings published by Castiglione et al. [8] and Rutgeerts et al. [9], who found SIBO in 23–25% of patients with CD, although their studies were conducted with much smaller numbers of patients. Castiglione et al. used the H_2_-lactulose breath test (HLBT) to screen for the presence of bacterial overgrowth in a group of 57 patients with either intact or surgically resected ileocecal valves [8]. The collective studied by Rutgeerts et al. included 61 patients who had not undergone prior surgery [9].

The H_2_-glucose breath test (HGBT) used in this study is reported in the literature to have a sensitivity of 62–93% and a specificity of 78–100% in detecting SIBO [1,3]. The H_2_-lactulose breath test (HLBT), however, which detects bacterial overgrowth by determining the oro-cecal transit time, shows a sensitivity of only 16.7–68% and a specificity of 44–70% [1,3]. Although some studies have suggested a possible superiority of the ^14^C-xylose breath test (XBT), which uses ^14^C as radioactive marker, compared to HGBT, these findings remain controversial and the sensitivity and specificity of the method have been reported at 60–70% and 40%, respectively [1,5,15]. Because it is simpler to administer, more cost-effective and does not expose the patient to radiation, the HGBT has become the non-invasive gold standard in the diagnosis of SIBO [1,3,5].

False positive HGBT findings may occur in the absence of SIBO in patients with accelerated gastric emptying or with an accelerated oro-cecal transit time [1,3,4]. False positive findings may also be returned in patients with prior extensive resection of the small bowel which leads to accelerated intestinal transit as part of a short-bowel syndrome [10]. A false negative HGBT may occur in patients receiving antibiotics or with induced reduction in intestinal bacterial load secondary to bowel preparation, e.g. prior to colonoscopy [3].

A more difficult problem relates to hydrogen non-producers, which make up about 10% of the population [1,3]. A non-producer status was determined in three patients (2%) in our study, which is low compared to the general population. On the other hand, a hydrogen non-producer status was not excluded in all cases by means of the HLBT but only in those patients reporting typical symptoms who did not exhibit a significant H_2 _increase in exhaled air.

The symptoms of SIBO include weight loss, diarrhea, meteorism, flatulence and abdominal pain. Correspondingly, patients in the present study with documented SIBO not only experienced significantly more frequent symptoms during the HGBT, but also reported a significantly higher stool frequency prior to HGBT. In addition, Crohn's patients who tested positive for SIBO also showed a significantly lower absolute body weight than did patients without SIBO.

CDAI served as the parameter expressing disease activity at the time of test administration. Patients with bacterial overgrowth exhibited on average only a trend to elevated levels (166.7 vs. 138.9; p = 0.098). A comparison of the prevalence of bacterial overgrowth in subgroups of patients with low (CDAI <150), moderate (CDAI = 150–300) and high (CDAI >300) disease activity showed no significant differences. This confirms data reported by Mishkin et al., who found no statistically significant correlation between CDAI and HGBT results in patients with CD [10].

Because stool frequency is an important factor in calculating CDAI, interpreting CDAI values in patients with SIBO is not always easy. The number of daily bowel movements was significantly higher in patients with SIBO than in those without bacterial overgrowth (5.9 vs. 3.7 bowel movements per day; p = 0.003). Since increased stool frequency is a symptom frequently reported in patients with SIBO, an increase in CDAI cannot be ascribed exclusively to an increase in inflammatory activity. We conclude that the CDAI in patients with CD complicated with SIBO is less useful to assess disease activity due to the potentially significant increase in stool frequencies by reasons other than CD activity.

Reported risk factors for the development of bacterial overgrowth in patients with CD include surgical procedures involving the gastrointestinal tract, especially ileocecal resection, as well as strictures or fistulae [3,8,10]. Surgery affecting the integrity of the ileocecal valve may be followed by changes in the bacterial colonization of the proximal ileum characterized especially by an increase in the proportion of Gram-negative species [2,7]. The removal of the physiological barrier represented by the ileocecal valve allows for a backwash of bacteria-laden intestinal contents from the colon into the small bowel [4,7].

Castiglione et al. have shown that the prevalence of SIBO is higher in patients with prior ileocecal resection than in those who have not undergone prior surgery (30% vs. 18%; [8]). None of the 61 patients in the study by Rutgeerts et al. had undergone prior surgery [9]. In the present study, prior ileocecal resection had been performed in 23 of 38 patients (60.5%) with documented SIBO. Furthermore, nearly half of all patients who had undergone either prior partial colectomy or multiple resective procedures affecting the small bowel showed evidence of SIBO, a rate that was significantly higher than that reported for the group of patients who had not undergone prior colectomy or multiple resective procedures. One reason might be a change, especially a reduction in motility in the bowel following surgery. This may be reflected in data reported by Castiglione et al., who found a prolongation of oro-cecal transit time in patients with CD compared to healthy controls [8]. This prolongation of oro-cecal transit time was especially pronounced in patients with prior ileocecal resection compared with Crohn's patients who had not undergone prior surgery. It is possible that narrowing of the intestinal lumen at anastomotic sites or subsequent recurrence of stenoses may contribute to stasis and prolonged transit of intestinal contents.

Fifteen patients in our study who had tested positive for SIBO had no history of prior surgical procedures. Castiglione et al. implicate entero-enteric fistulae and strictures as possible etiological factors in the development of SIBO in patients who have not undergone prior surgery [8]. Mishkin et al. describe bacterial overgrowth as an indicator for strictures in patients with CD [10]. In their study, 24 of 25 patients (96%) with radiologically confirmed strictures exhibited SIBO. Only nine of these patients had a prior history of ileocecal resection. Stasis of the intestinal contents secondary to strictures has been discussed as a cause of bacterial overgrowth. In the Rutgeerts trial, of 14 patients with evidence of SIBO, six suffered from radiologically confirmed strictures or fistulae [9]. In the remaining patients, bacterial overgrowth was believed secondary to the altered motility of the gastrointestinal tract occurring in patients with CD.

Strictures and fistulae in patients with chronic inflammatory bowel diseases are often asymptomatic [10]; hence, it is difficult to make statements on the presence of strictures or fistulae without radiological or endoscopic findings. Because no radiological data to exclude fistulae were documented at the outset of our study, no statement can be made regarding the presence of entero-enteric fistulae in our patient collective. However, dysfunction of Bauhin's valve secondary to chronic inflammation in the terminal ileum could also be considered a potential promoting factor for SIBO [9]. Thus, patients with involvement of the neo- or terminal ileum might be considered at higher risk for bacterial overgrowth. Although the findings of the present study showed a strong trend in this direction, data did not reach the level of statistical significance.

The treatment of bacterial overgrowth includes both surgical procedures aimed at relieving stasis by means of stricturoplasty or resection of a stenotic bowel segment and antibiotic therapy [6]. For example, antibiotic therapy with norfloxacin or amoxicillin/clavulanate was shown to be significantly more effective than placebo in the treatment of SIBO in patients without inflammatory bowel disease [10]. Castiglione et al. reported therapeutic success using metronidazole or ciprofloxacin in patients with CD [13]. But in many cases, antibiotic treatment may not show a long-lasting effect because predisposing factors allowing recurrence remain present and repeated or periodic courses of antibiotics or a combination of antibiotics are necessary. Therapy of SIBO with probiotics is being investigated in running studies.

## Conclusion

In conclusion, bacterial overgrowth represents a complication of CD that is often overlooked but may have significant clinical relevance for patients mimicking acute flare. It may lead to malnutrition or produce a complex of clinical symptoms that may be impossible to distinguish from patients' underlying disease. Typically, patients' clinical symptoms are ascribed to inflammatory activity in the bowel and treated with anti-inflammatory regimens such as topical or systemic steroids. Because patients with chronic inflammatory bowel diseases often experience malabsorption and gastrointestinal complaints, recognition and specific therapy of SIBO can be of decisive importance and must always be considered in the management of these patients.

## Abbreviations

BMI: body mass index; CD: Crohn's Disease; CDAI: Crohn's Disease Activity Index; CFU: colony forming units; f: female; g: gram; H2: hydrogen gas; HBT: Hydrogen Breath Test; HGBT: Hydrogen Glucose Breath Test; HLBT: Hydrogen Lactulose Breath Test; m: male; ml: mililiter; ppm: parts per million; SIBO: small intestinal bacterial overgrowth.

## Grant support, competing interests and financial disclosures

The authors declare that they have no competing interests.

## Authors' contributions

JK, US and ChvonT contributed to conception and design, acquisition, analysis and interpretation of data, drafted and revised the manuscript; GA, RM and MR contributed to conception and design and revised the manuscript critically for important intellectual content; all authors have given final approval of the version to be published.

## Pre-publication history

The pre-publication history for this paper can be accessed here:

http://www.biomedcentral.com/1471-230X/9/61/prepub
